# Restrained Eating and Disinhibited Eating: Association with Diet Quality and Body Weight Status Among Adolescents

**DOI:** 10.3390/nu16213601

**Published:** 2024-10-23

**Authors:** Joanna Kowalkowska, Jadwiga Hamulka, Lidia Wadolowska, Magdalena Górnicka, Ewa Czarniecka-Skubina, Krystyna Gutkowska

**Affiliations:** 1 Department of Human Nutrition, Faculty of Food Science, University of Warmia and Mazury in Olsztyn, 45F Sloneczna Street, 10-718 Olsztyn, Poland; joanna.kowalkowska@uwm.edu.pl (J.K.); lidia.wadolowska@uwm.edu.pl (L.W.); 2 Department of Human Nutrition, Institute of Human Nutrition Sciences, Warsaw University of Life Sciences (SGGW-WULS), 166 Nowoursynowska Street, 02-787 Warsaw, Poland; magdalena_gornicka@sggw.edu.pl; 3 Department of Food Gastronomy and Food Hygiene, Institute of Human Nutrition Sciences, Warsaw University of Life Sciences (SGGW-WULS), 166 Nowoursynowska Street, 02-787 Warsaw, Poland; ewa_czarniecka-skubina@sggw.edu.pl; 4 Department of Food Market and Consumption Research, Institute of Human Nutrition Sciences, Warsaw University of Life Sciences (SGGW-WULS), 166 Nowoursynowska Street, 02-787 Warsaw, Poland; krystyna_gutkowska@sggw.edu.pl

**Keywords:** eating behaviour, eating styles, cognitive restraint, uncontrolled eating, emotional eating, disinhibited eating, body mass index, obesity, teenagers

## Abstract

**Background/Objectives**: Problematic eating behaviours may affect food consumption and, therefore, body weight. However, these associations have not been well understood, especially among adolescents. The aim of the study was to evaluate the associations of restrained eating and disinhibited eating with diet quality and body weight status among adolescents. **Methods**: A cross-sectional study was conducted among 1450 primary school students aged 11–13 years (52% girls). Dietary data were collected using the food frequency questionnaire (SF-FFQ4PolishChildren^®^). Two diet quality scores were evaluated: (i) the pro-Healthy Diet Index (pHDI), which included vegetables, fruit, dairy products, and fish, and (ii) the non-Healthy Diet Index (nHDI), which included fast food, sweets, sweetened beverages, and energy drinks. Based on the Three-Factor Eating Questionnaire, two eating styles were identified: restrained eating (RE) and disinhibited eating (DE). Body weight status was evaluated using the body mass index (BMI) z-score and the waist-to-height ratio (WHtR). Spearman’s correlation coefficient and logistic regression analysis were used to assess the association between eating styles, diet quality, and body weight status. **Results**: Restrained eating was negatively correlated with nHDI (r = −0.178, *p* < 0.001) and positively correlated with the BMI z-score (r = 0.253, *p* < 0.001) and WHtR (r = 0.197, *p* < 0.001). Disinhibited eating was positively correlated with nHDI (r = 0.232, *p* < 0.001). Among adolescents with different RE and DE levels, significant differences in the mean nHDI, BMI z-score, and WHtR were found. Compared to adolescents with ‘low RE & DE’, those with ‘low RE & higher DE’ were more likely to fall in the upper than bottom tertile of nHDI (odds ratio (OR) =1.90, 95% CI: 1.29–2.81). Adolescents with ‘higher RE & low DE’ were less likely to be underweight (OR = 0.17, 95% CI: 0.06–0.49) and more likely to be overweight (OR = 2.02, 95% CI: 1.41–2.91) and to have abdominal obesity (OR = 1.79, 95% CI: 1.13–2.82). **Conclusions**: The findings suggest that both eating styles may be predictors of diet quality among adolescents. Body weight status was related to restrained eating, which seems to characterise mainly adolescents with overweight or obesity.

## 1. Introduction

Overweight and obesity in childhood is a growing public health problem. Globally, one in five children and adolescents aged 5–19 were overweight or obese in 2022 (20.7% of boys, 18.7% of girls), which is 2.5 times higher than in 1990 [1]. The prevalence of obesity has quadrupled since 1990, and in 2022 amounted to 8.2%. In Poland, 23% of children and adolescents aged 5–19 were overweight or obese in 2022 (24.7% of boys and 21.2% of girls) [2]. In turn, thinness in 2022 occurred in 9.6% of children and adolescents aged 5–19 worldwide (10.8% of boys, 8.3% of girls) and decreased compared to 1990 (13.5%). In Poland, thinness amounted to 2.9% of this age group in 2022 [2].

An abnormal body weight results from an energy intake imbalance due to genetic predispositions, psychosocial factors, and/or an obesogenic environment (e.g., limited availability of healthy food due to high prices) [1]. Adolescents are becoming more independent from their parents than younger offspring and are particularly susceptible to adverse dietary behaviours due to the influence of peers, mass media, and various dietary trends [3]. The diet quality and body weight in children and adolescents are determined by many factors, such as sex, age, place of residence (rural/urban areas), economic situation of the family, nutrition knowledge, physical activity, and screen time [1,3,4,5]. The diet quality is typically assessed using various diet quality scores developed based on predefined criteria following dietary recommendations [6,7]. This holistic approach is considered to have advantages over the assessment of single dietary characteristics (i.e., foods or nutrients) in nutrition epidemiology. However, the application of predefined diet quality scores in epidemiological studies requires several decisions by researchers related to the number and type of components to include in the score as well as the method of scoring [6,7,8]. The most frequently used diet quality scores were the Mediterranean diet (MD) scores, Healthy Eating Index (HEI) and its adaptations, and Dietary Approaches to Stop Hypertension (DASH) scores [8]. These overall healthy diet scores focused on higher consumption of fruit and vegetables, low-fat dairy products, fish, pulses, and whole grains, but lower consumption of red and processed meat and sweet and/or fatty foods. It was stated that a high energy-dense, low-fibre, and high-fat diet was associated with excess adiposity in childhood [9].

Problematic eating behaviours include restrained eating, emotional eating, and uncontrolled eating (also called external eating) [10,11,12]. These eating behaviours are also referred to as problematic eating styles [13,14]. Restrained eating (or dietary restraint) reflects a cognitive effort to limit food consumption to control body weight, including strategies such as using small portions, avoiding high-energy and fatty foods, and stopping eating before reaching satiety [11,15,16]. In some studies, this eating style has been related to a healthier diet [11,17]. However, paradoxically, higher restraint in eating can increase the risk of overeating, for example, in stressful situations when self-control can be lower [15,18]. Some studies showed that restrained eating can be a predictor of weight gain [15,19]. Overall, the results on the association between restrained eating and both dietary intake and body mass index (BMI) are still inconsistent [11]. Emotional eating is food consumption in response to emotions (negative or positive), which can lead to overeating and body weight gain [20,21]. Food consumption is used as a maladaptive strategy to cope with emotions such as sadness or anxiety. Emotional eaters to feel better usually choose energy-dense and palatable foods (e.g., sweet or salty snacks, fatty foods) [22]. Uncontrolled eating involves overeating due to external triggers, such as food availability and palatability, as well as social factors (e.g., the presence of other people), instead of relying on internal cues like hunger and satiety levels [10,11,23,24]. This eating style reflects eating large amounts of food without feeling in control and has also been linked to a higher BMI [10,11]. Disinhibited eating includes elements of both uncontrolled eating and emotional eating and reflects a tendency to overeat due to external and emotional cues [11]. This eating style is characterised by hedonic food choices (i.e., high in sugar and/or fat) and can lead to poor diet quality [11,17]. Disinhibited eating may result in overweight and increase the risk of eating disorders (e.g., binge eating disorder) [11].

Problematic eating styles among Polish children and/or adolescents have been evaluated in a limited number of studies [12,25,26,27]. The association between the problematic eating styles and BMI of children and/or adolescents was inconsistent based on the research conducted in Poland [12] as well as in other countries [10,28,29,30,31]. The variations in the body weight of people with different levels of restraint and disinhibition in eating, to some extent, may be explained by diet quality [11]. Only a few studies have focused on the eating styles of children and/or adolescents and their association with food consumption worldwide [17,25,32,33]. One study assessed the relationship between two eating styles (restrained and uncontrolled eating) and the frequency of snack consumption among Polish children and adolescents [25]. To the best of the authors’ knowledge, the relationship between problematic eating styles and diet quality in Poland was investigated in research carried out among adults only [14]. Difficulties in regulating emotions during adolescence, as well as following different dietary trends, may contribute to the development of problematic eating styles and affect adolescents’ dietary behaviours and body weight [34]. However, there is a lack of holistic research on the association between problematic eating styles, diet quality, and body weight in adolescents. Understanding these relationships is crucial to developing effective strategies to promote healthy eating and interventions to prevent obesity among adolescents. Therefore, the study aimed to evaluate the associations of restrained eating and disinhibited eating with diet quality and body weight status among adolescents. These relationships were assessed for each eating style separately as well as for groups of adolescents with different levels of both restrained and disinhibited eating.

## 2. Materials and Methods

### 2.1. Study Design and Participants

A cross-sectional study was conducted among primary school students aged 11–13 years. Pooled data gathered in 2015–2016 within two projects, i.e., the ‘ABC of Healthy Eating’ project and the ‘ABC of Healthy Eating—ABC of Kids’ Nutrition’ project, were used. The data were collected by well-trained researchers from nine Polish universities. A convenient sample selection was used. Participants were recruited in primary schools from all macro-regions of Poland, covering rural and urban areas and located at a convenient distance from the academic centres (<50 km). Students of 4th and 5th grade classes were invited to participate in the study. In total, 1678 students were recruited whose parents or legal guardians gave written consent to participate in the study. The obtained data were then verified due to inclusion criteria, and 109 adolescents were excluded. The study protocol was described in more detail previously [35]. Furthermore, 119 adolescents were excluded from the statistical analysis in the present study due to the missing data regarding eating styles (n = 26), diet quality (n = 7), body weight status (n = 75), and other variables (n = 11). The final sample included 1450 adolescents aged 11–13 years (52% girls).

The data were collected using a short form of a food frequency questionnaire (SF-FFQ4PolishChildren^®^) developed by Kowalkowska, Wadolowska, and Hamulka [35]. The SF-FFQ4PolishChildren^®^ has been validated among children (6–10 years old) and adolescents (11–15 years old) [36]. The questionnaire in a paper-and-pencil format was self-administered by adolescents in the classrooms. Well-trained researchers provided information about the study, explained how to complete the questionnaire, answered any respondents’ questions, and also conducted anthropometric measurements of the adolescents.

### 2.2. Problematic Eating Styles

Problematic eating styles were assessed with the Three-Factor Eating Questionnaire (TFEQ) [37], adapted and validated among Polish adolescents by Dzielska et al. [26]. The Polish version of the TFEQ consisted of 13 statements. However, due to a weak understanding of three statements during the pilot study, it was decided to use a shorter version of this questionnaire (10 statements) [35]. Based on the principal component analysis (PCA), two eating styles were then identified: (i) restrained eating (RE; 3 statements) and (ii) disinhibited eating, including both emotional eating and uncontrolled eating (DE; 7 statements). Respondents responded to the statements by choosing one of four answers: definitely not (0 points), rather not (1 point), rather yes (2 points), and definitely yes (3 points). The scores of RE and DE were calculated by summing the points assigned to the answers given by respondents and expressed in the range from 0 to 100 points. The internal consistency for both RE and DE was satisfactory (Cronbach’s alpha was 0.60 and 0.75, respectively). Four groups of respondents were distinguished based on the median values of RE (44.4 points) and DE (28.6 points): (i) low RE and DE, (ii) higher RE and low DE, (iii) low RE and higher DE, and (iv) higher RE and DE.

### 2.3. Diet Quality

Diet quality was assessed with two diet quality scores: (i) a pro-Healthy Diet Index (pHDI) and (ii) a non-Healthy Diet Index [35], which have been validated among children and adolescents aged 6–15 years [36]. The pHDI was developed based on the consumption frequency of four food groups: vegetables, fruit, dairy products, and fish. The nHDI was developed based on the consumption frequency of four food groups: fast food, sweets, sweetened beverages, and energy drinks. The respondents evaluated their habitual frequency of food consumption during the last 12 months by choosing one of seven categories, converted into daily consumption frequencies for further analysis: never or almost never (0 times/day), rarely than once a week (0.06), once a week (0.14), 2–4 times/week (0.43), 5–6 times/week (0.79), every day (1), and several times a day (2). The diet quality scores were calculated by summing the daily consumption frequencies (times/day) and expressed in the range from 0 to 100 points. The respondents were classified into categories created based on the tertile distribution of each diet quality score as follows: (i) pHDI: bottom tertile (<21.3 points), middle tertile (21.4–32.2 points), and upper tertile (≥32.3 points); and (ii) nHDI: bottom tertile (<7.8 points), middle tertile (7.9–15.9 points), and upper tertile (≥16.0 points).

### 2.4. Body Weight Status

The measurements of height (cm), body weight (kg), and waist circumference (WC, cm) were taken in line with the International Standards for Anthropometric Assessment [38]. The same type of professional equipment was used across all research centres, i.e., a portable stadiometer (SECA 220, seca Gmbh & Co. KG, Hamburg, Germany) for height measurement, an electronic digital scale (SECA 799, seca Gmbh & Co. KG, Hamburg, Germany) for weight measurement, and a stretch-resistant tape (SECA 201, seca Gmbh & Co. KG, Hamburg, Germany) for WC measurement [35]. The respondents were measured in light clothing and without shoes. Body weight status was evaluated using the body mass index (BMI) and waist-to-height ratio (WHtR). The z-scores for adolescent BMI were calculated based on the Polish standards developed in the range of the OLAF project [39]. The body weight status of respondents was then categorised as follows: underweight (BMI z-score < −1 SD), normal weight (−1 SD ÷ 1 SD), and overweight (>1 SD). The prevalence of abdominal obesity was classified based on the WHtR ≥ 0.5 [40].

### 2.5. Covariates

The data regarding demographic, socioeconomic, and lifestyle factors were collected using the questionnaire as well and included the following: sex (boy/girl), age (in years), place of residence (rural/urban), family affluence, nutrition knowledge, physical activity, and screen time [35].

The family affluence scale (FAS) was utilised to assess the socioeconomic status of the respondent’s family. The FAS consisted of four questions regarding having one’s own bedroom, travelling on holidays with a family, and the number of cars and computers owned by a family [41]. Answers were scored from 0 to 2 points. The FAS score was calculated by summing the points obtained by a respondent (a possible range: 0–7 points), and the higher the score, the better the socioeconomic status of the adolescent’s family. Respondents were classified into FAS categories, created based on quartile distribution: low (<25th quartile; 0–4 points), moderate (5–6 points), and high (≥75th quartile; 7 points).

Nutrition knowledge (NK) was evaluated based on eighteen questions [35]. For each correct answer, respondents received 1 point. The NK score was calculated by summing the points obtained by a respondent (possible range: 0–18 points). The respondents were classified into NK levels as follows: low (0–5 points), moderate (6–12 points), and high (13–18 points). Due to the small number of respondents with high NK (n = 22), both moderate and high NK levels were combined for further analysis.

The lifestyle of adolescents was evaluated with three questions regarding physical activity at school, physical activity in leisure time, and screen time [35]. The respondents described their physical activity at school or leisure time by choosing one of three categories: low, moderate, and vigorous. Many examples for each answer were given to facilitate the assessment of the adolescent’s own physical activity, and the total physical activity was evaluated based on both PA questions. The total PA score was in the range of 0 to 5 points, i.e., 0 points were assigned for respondents with low PA in both questions, and 5 points were assigned for respondents with high PA in both questions. Finally, the respondents were classified into PA levels as follows: low (0–1 point), moderate (2–4 points), and high (5 points). Screen time was assessed using a question with six categories to choose from: <2 h/day, 2 to <4 h/day, 4 to <6 h/day, 6 to <8 h/day, 8 to <10 h/day, and ≥10 h/day, which were scored from 0 to 5 points, respectively. Due to the small number of respondents in higher categories, three ST categories were considered in further analysis: <2 h/day, 2 to <4 h/day, and ≥4 h/day. The details were described previously [35,42].

### 2.6. Statistical Analysis

The normality of the distribution of continuous variables was tested using the Kolmogorov–Smirnov test. The means and standard deviations (SD) of age, the FAS, the NK score, the PA score, the ST score, eating styles (RE, DE), the diet quality scores (pHDI, nHDI) and the daily consumption frequency of their components (8 food items), the BMI z-score, and the WHtR were calculated. The mean values were compared between boys and girls with the Mann–Whitney test and between four RE and DE levels with the Kruskal–Wallis test and post-hoc analysis. Differences in the percentage distribution of the categorical variables were verified with the chi^2^ test with Yates’ correction if necessary.

Spearman’s correlation coefficients between eating styles (RE, DE) and diet quality scores (pHDI, nHDI), body weight measures (BMI z-score, WHtR), as well as covariates (FAS, NK score, PA score, ST score) were calculated. The correlations were interpreted as follows: negligible (0.01–0.09), weak (0.10–0.29), moderate (0.30–0.49), strong (0.50–0.69), very strong (0.70–0.89), and perfect (0.90–1.00) [43].

Logistic regression was applied to evaluate the association between (i) the independent variable—the eating style levels (low RE and DE as the reference group)—and (ii) outcomes: diet quality scores (in tertiles; bottom tertile as the reference group) and body weight status (the reference groups: normal weight (BMI z-score: −1 SD ÷ 1 SD)), and lack of abdominal obesity (WHtR < 0.5). The odds ratios (ORs) and 95% confidence intervals (95% CIs) were calculated. The ORs were adjusted for the following confounders: sex (boys/girls), age (years), place of residence (rural/urban), FAS (points), the NK score, the PA score, the ST score, and the BMI z-score (in the analyses for diet quality scores). The significance of ORs was tested by Wald’s statistics.

*p*-values below 0.05 were considered statistically significant in all analyses. The statistical analyses were performed with Statistica 13 (TIBCO Software Inc., Tulsa, OK, USA; StatSoft Polska, Cracow, Poland) and PS IMAGO PRO 9.0 on the IBM SPSS Statistics 29 analytical engine (Predictive Solutions, Cracow, Poland).

## 3. Results

### 3.1. Participant Characteristics

The characteristics of participants in the total sample and sex groups are presented in Table 1. Boys (48.3% of the sample) scored lower in NK and the pHDI, and higher in lifestyles (PA score, ST score), the nHDI, and the WHtR than girls. Similarly, the percentage distribution between boys and girls varied for NK, PA, ST, tertiles of the pHDI, tertiles of the nHDI, BMI z-score categories, and the WHtR categories. The mean pHDI was 27.5 points among boys and 29.2 points among girls (*p* = 0.007), while the mean nHDI was 15.0 points and 13.1 points, respectively (*p* < 0.001). There was a higher proportion of boys than girls with abdominal obesity (16.3% vs. 8.5%, *p* < 0.001, respectively).

There was no significant difference in the mean values of both eating styles or the percentage distribution of RE and DE levels between boys and girls. In the total sample, the mean RE was 48.2 points, and the mean DE was 31.5 points. The proportion of adolescents with ‘low RE & DE’ was 24.6%, with ‘higher RE & low DE’ was 26.6%, with ‘low RE & higher DE’ was 27.6%, and with ‘higher RE & DE’ was 21.2%. Participant characteristics across the RE and DE levels are shown in Table S1.

### 3.2. Eating Styles and Demographic, Socioeconomic, and Lifestyle Factors

Both eating styles (RE and DE) were negatively correlated (r = −0.137, *p* < 0.001; Table 2). Restrained eating (RE) was positively correlated with the FAS (r = 0.070, *p* = 0.007) and PA score (r = 0.111, *p* < 0.001) and negatively correlated with the ST score (r = −0.071, *p* = 0.007). On the contrary, disinhibited eating (DE) was negatively correlated with the NK score (r = −0.073, *p* = 0.005) and PA score (r = −0.138, *p* < 0.001) and positively correlated with the ST score (r = 0.197, *p* < 0.001).

Differences in the mean values of each eating style by the demographic, socioeconomic, and lifestyle factors are presented in Table S2. The mean RE was higher among adolescents from rural than urban areas (*p* = 0.048), with moderate or high PA than with low PA (*p* < 0.001), and adolescents with ST < 2 h/day than those with ST ≥ 4 h/day (*p* = 0.025). The mean DE was higher among adolescents with low NK than with moderate/high NK (*p* = 0.010), with low PA than those with higher PA levels (*p* < 0.001), and with ST ≥ 4 h/day than those with lower ST (*p* < 0.001).

When comparing groups of adolescents with different RE and DE levels, significant differences were found in the mean values of the FAS, NK score, PA score, and ST score as well as in the percentage distribution of these variables, except for the FAS levels (Table S1). Adolescents with ‘higher RE & low DE’ scored the highest in the FAS, NK score, and PA score, and the lowest in ST scores (5.5, 6.3, 3.9, and 0.6 points, respectively). These results differed significantly from those obtained for adolescents with ‘low RE & higher DE’ who scored the lowest in the FAS, NK score, and PA score, and the highest in ST scores (5.2, 5.7, 3.4, and 1.0 points, respectively). There were no significant differences by sex, age, or place of residence.

### 3.3. Eating Styles and Diet Quality

Restrained eating (RE) was negatively correlated with the nHDI (r = −0.178, *p* < 0.001), while disinhibited eating (DE) was positively correlated with the nHDI (r = 0.232, *p* < 0.001) (Table 2). There were no significant correlations between both eating styles and the pHDI. These findings were confirmed by comparing the mean values of both eating styles across the tertiles of the diet quality scores (Table S2).

When comparing groups of adolescents with different RE and DE levels, significant differences in the mean values of the nHDI (*p* < 0.001) but not the pHDI (*p* = 0.351) were found (Table 3). Among adolescents with ‘low RE & higher DE’, the mean nHDI was 17.4 points, significantly higher than in the other groups (the range of differences: 3.4 to 6.8 points). The lowest nHDI (10.6 points) was noted among adolescents with ‘higher RE & low DE’. The frequency of consumption (times/day) differed across RE and DE levels for all the nHDI components (*p* < 0.001 for all). For example, adolescents with ‘low RE & higher DE’ compared to those with ‘higher RE & low DE’ had a significantly higher frequency of consumption of fast food (0.17 vs. 0.08 times/day), sweets (0.65 vs. 0.41 times/day), sweetened beverages (0.51 vs. 0.33 times/day), and energy drinks (0.06 vs. 0.03 times/day). Considering the pHDI components, differences were noted for vegetables (*p* = 0.034), fruit (*p* = 0.016), and fish (*p* = 0.049). For example, adolescents with ‘higher RE & low DE’ had a significantly higher frequency of consumption of fruit than those with ‘higher RE & DE’ (0.77 vs. 0.65 times/day). Differences in the percentage distribution were observed for the nHDI tertiles only (*p* < 0.001).

Compared to adolescents with ‘low RE & DE’ (the reference group), those with ‘higher RE & low DE’ were more likely to fall in the middle tertile of the pHDI (OR_crude_ = 1.50, 95% CI: 1.05–2.14) and less likely to fall in the upper tertile of the nHDI (OR_crude_ = 0.56, 95% CI: 0.39–0.80) than in the bottom tertile (Table 4). On the contrary, adolescents with ‘low RE & higher DE’ were more likely to fall in the middle tertile of the nHDI (OR_crude_ = 1.51, 95% CI: 1.04–2.20; OR_adj_ = 1.49, 95% CI: 1.02–2.19) and the upper tertile of the nHDI (OR_crude_ = 2.06, 95% CI: 1.44–2.95; OR_adj_ = 1.90, 95% CI: 1.29–2.81). There was no significant association between adolescents with ‘higher RE & DE’ and diet quality scores.

### 3.4. Eating Styles and Body Weight Status

Restrained eating (RE) was positively correlated with the BMI z-score (r = 0.253, *p* < 0.001) and WHtR (r = 0.197, *p* < 0.001) (Table 2). There were no significant correlations between disinhibited eating (DE) and both the BMI and WHtR. These findings were confirmed by comparing the mean values of both eating styles across the BMI and WHtR categories (Table S2).

When comparing groups of adolescents with different RE and DE levels, significant differences in the mean values of the BMI z-score (*p* < 0.001) and WHtR (*p* < 0.001) were found (Table 3). The BMI z-scores obtained among adolescents with ‘higher RE & low DE’ (0.63) and ‘higher RE & DE’ (0.53) were significantly higher than noted in two other groups, i.e., adolescents with ‘low RE & DE’ (0.17) and those with ‘low RE & higher DE’ (0.24). Similarly, a higher WHtR was found among adolescents with ‘higher RE & low DE’ (0.44) and ‘higher RE & DE’ (0.44) than those from two other groups (0.42 for each). Differences in the percentage distribution were found for both the BMI (*p* < 0.001) and WHtR (*p* = 0.048) as well. The highest proportion of adolescents with overweight (BMI z-score > 1 SD) and abdominal obesity (WHtR ≥ 0.5) was observed in the ‘higher RE & low DE’ group (30.1% and 15.5%, respectively).

Compared to adolescents with ‘low RE & DE’ (the reference group), those with ‘higher RE & low DE’ were less likely to be underweight (OR_crude_ = 0.18, 95% CI: 0.06–0.51; OR_adj_ = 0.17, 95% CI: 0.06–0.49) and more likely to be overweight (OR_crude_ = 1.80, 95% CI: 1.27–2.55; OR_adj_ = 2.02, 95% CI: 1.41–2.91) than to be normal weight (Table 4). Those adolescents were more likely to have abdominal obesity as well (OR_crude_ = 1.59, 95% CI: 1.02–2.46; OR_adj_ = 1.79, 95% CI: 1.13–2.82). Adolescents with ‘higher RE & DE’ were more likely to be overweight (OR_crude_ = 1.77, 95% CI: 1.23–2.56; OR_adj_ = 1.73, 95% CI: 1.19–2.52).

## 4. Discussion

The findings of this study suggest that both eating styles may be predictors of diet quality among adolescents, especially regarding unhealthy characteristics. Body weight status was related to restrained eating.

Both problematic eating styles (RE and DE) were negatively correlated among Polish adolescents, which is in line with the other research carried out among adolescents and adults. The negative correlation between restrictive eating and uncontrolled eating (r = −0.125) was also found in the other study conducted among Polish children and adolescents [25]. Similarly, cognitive restraint and uncontrolled eating were negatively correlated among adolescents from the UK (r = −0.20) [10]. Other research showed that cognitive restraint was negatively correlated with the disinhibition measured with the TFEQ (r = −0.20) and the uncontrolled eating measured with the TFEQ-R18 (r = −0.34) in middle-aged obese people [37].

In the present study, there were no differences between adolescent boys and girls in both eating style scores and across the groups of adolescents with different RE and DE levels. However, the research conducted among Polish children and adolescents aged 8–16 years showed significant sex and age differences in restrictive eating and emotional eating but not in uncontrolled eating measured with the TFEQ-13 [12]. Restrictive eating was higher in girls than boys, irrespective of the age group, while emotional eating was higher in girls from the older group only (aged 12–16 years). Moreover, both eating styles were higher in adolescent girls (12–16 years old) than the younger ones (8–11 years old) [12]. A study carried out in France showed significant sex differences in the eating styles in people aged 14–22 years, i.e., cognitive restraint and emotional eating were higher among girls, while uncontrolled eating was higher among boys [44]. However, the obtained results can differ depending on the questionnaire used to assess the eating styles as well as the perspective of children and their parents [12]. Furthermore, parents’ eating style can affect children’s eating style. A study carried out in parent–adolescent dyads showed positive correlations between the emotional eating level in parents (mothers and fathers) and their sons, and the uncontrolled eating or the emotional eating levels in mothers and their daughters, but there was no significant correlation for cognitive restraint levels in parent–offspring dyads [44].

The current findings showed that restrained eating was positively related to family affluence, while disinhibited eating was negatively associated with nutrition knowledge among adolescents. However, the findings regarding the association between socioeconomic status (SES) and the eating styles of different population groups were inconsistent across countries [31,45,46,47]. Similar to the present study, a higher family income was positively related to restrained eating, but there was no significant association with emotional eating or external eating among adolescents in China [31]. However, a study conducted among university students in Poland showed no significant association between SES and eating styles measured with the TFEQ-13 [45]. Research carried out among adults in Germany demonstrated positive but weak correlations between SES and the three eating styles [46]. A study conducted in France showed that uncontrolled eating was lower in people with a high SES, but there were no significant associations with cognitive restraint and emotional eating [47]. However, there has been little research on the association of eating styles with nutrition knowledge. It has been shown that nutritional knowledge was positively related to restraint eating and negatively related to the hunger dimension measured with the TFEQ in female university students satisfied with their weight [48].

The current findings showed that restrained eating was positively related to physical activity (PA) but negatively related to screen time. On the contrary, disinhibited eating was negatively associated with physical activity but positively associated with screen time. There have only been a few studies, including the associations of eating styles with lifestyle factors, conducted among adolescents [49,50] or adults [45,48]. Similarly, a study carried out among Malaysian adolescents showed a positive association between emotional eating and screen time [50]. A 6-month exercise intervention decreased external eating and food cravings among adolescents with overweight and obesity in Canada [49]. Research carried out among Polish university students showed that PA was positively associated with cognitive restraint among females and negatively related to emotional eating among males [45]. Similar results were found in another study conducted among female university students dissatisfied with their weight [48]. It was found that the exercise level was positively related to cognitive restraint but negatively associated with disinhibition measured with the TFEQ. Females with higher disinhibited eating usually had more sedentary behaviours [51].

The current findings demonstrated that both eating styles may be predictors of diet quality among adolescents, especially regarding unhealthy characteristics. The non-Healthy Diet Index (nHDI) was negatively related to restrained eating but positively related to disinhibited eating. Considering adolescents with different RE and DE levels, those with ‘low RE & higher DE’ were about 1.5 times more likely to fall in the middle tertile of the nHDI and 1.9 times more likely to fall in the upper tertile of the nHDI, compared to adolescents with ‘low RE & DE’ (after adjustment for other factors). Furthermore, the frequency of consumption differed across the groups of adolescents with different RE and DE levels for all nHDI components (i.e., fast food, sweets, sweetened beverages, and energy drinks), with the most frequent unhealthy food consumption noted in adolescents with ‘Low RE & higher DE’. Similar associations of disinhibition with food choices have been found in other studies worldwide [17,25,32,51]. Disinhibition was suggested as the best predictor of food consumption [51]. Moreover, high disinhibition was related to higher food consumption, regardless of the restraint level. People characterised by high disinhibition were more likely to consume sweets, processed meat, high-fat and/or high-salt food, sweetened beverages, and coffee [17,51]. In another study carried out among Polish children and adolescents, uncontrolled eating was positively correlated with high-calorie snacks (e.g., sweets, fast food, salty sticks) [25]. Research conducted among French adolescents and young adults showed that higher uncontrolled eating was related to the higher consumption of French fries and cakes or pastries [32]. Moreover, the diet quality of children was influenced by the disinhibition level of their mothers [17]. Higher mothers’ disinhibition was related to higher fast-food consumption (hamburgers, French fries) and a lower consumption of some vegetables (e.g., cabbage, carrots, spinach). Thus, the children’s diet was characterised by a higher intake of energy, protein, fat, cholesterol, phosphorus, and sodium [17]. Therefore, disinhibited eating can lead to poorer diet quality and overweight affecting children’s health. Research carried out among Polish adolescents showed high stress and low emotional support as risk factors of uncontrolled eating, while a positive attitude towards school was indicated as a protective factor [27].

The present study showed that the pro-Healthy Diet Index (pHDI) was not significantly associated with either of the two problematic eating styles among Polish adolescents. Adolescents with ‘higher RE & low DE’ were 1.5 times more likely to fall in the middle tertile of the pHDI, but after adjustment for other factors, this association was not significant. Moreover, there was no significant association between adolescents with ‘higher RE & DE’ and diet quality scores. Considering the pHDI components, differences across the groups of adolescents with different RE and DE levels were noted for vegetables, fruit, and fish, with the most frequent consumption of fruit and fish observed in adolescents with ‘Higher RE & low DE’. Based on the literature, it was expected that restrained eating would be related to a somewhat healthier diet [11]. However, the findings regarding the effect of restrained eating on diet quality were inconclusive. Research conducted on French adolescents and young adults showed that higher cognitive restraint was related to a higher consumption of green vegetables and lower consumption of sugar and confectionery, fats, French fries, cakes and pastries, and sweet beverages [32]. Females with higher cognitive restraint had a significantly lower energy intake. However, contrary to middle-aged adults, adolescents and young adults with high cognitive restraint were characterised by a lower consumption of several energy-dense foods rather than a higher consumption of healthy food [32]. Moreover, higher mothers’ cognitive restraint was correlated with a higher consumption of ice cream, fruit juices, vegetables, tuna, and shellfish, as well as a higher intake of vitamin C [17]. Nevertheless, restrained eating may also lead to disinhibition and binge eating, especially during periods of increased psychological stress [15,16,18]. Thus, both restrained and disinhibited eating can contribute to the development of eating disorders [11]. Research conducted among Polish adolescents showed a high BMI, female gender, and low self-esteem as risk factors for cognitive restraint [27].

In the present study, body weight status was related to restrained eating. Interestingly, both measures of body weight status (i.e., the BMI z-score and WHtR) were positively related to restrained eating but not to disinhibited eating. Furthermore, a higher BMI z-score and WHtR were found among adolescents from the groups characterised by higher RE, irrespective of the DE level. Adolescents with ‘higher RE & low DE’ were almost six times less likely to be underweight but about two times more likely to be overweight and about 1.8 times more likely to have abdominal obesity compared to adolescents with ‘low RE & DE’ (after adjustment for other factors). Those with ‘higher RE & DE’ were about 1.8 times more likely to be overweight. In line with the current results, a positive association between restrained eating and the BMI was also found in adolescents from the UK [10], adolescents from China [31], and adolescent girls from the USA [29]. Rigid control was positively correlated with the BMI z-score in adolescent girls as well as in the total sample of adolescents from Canada [30]. Other research carried out among Polish children and adolescents has confirmed the positive correlation of restrained eating with the BMI [12,26] and shown a positive association with the prevalence of obesity [25]. Cognitive restraint was also higher among obese adolescents than those with normal weight in French adolescents [52]. Moreover, cognitive restraint was associated with body weight dissatisfaction in normal-weight adolescents. People with excessive body weight can exhibit higher cognitive restraint as a strategy to control body weight. On the other hand, paradoxically, higher restrained eating can lead to overeating and be a predictor of weight gain [15,19].

Surprisingly, disinhibited eating was not associated with adolescents’ body weight status. According to the literature review, disinhibition was related to a higher BMI, body weight, body fat, and weight regain after losing weight [11]. However, the overall moderate intensity of disinhibited eating in adolescents suggests that the more frequent food consumption may be associated with the pubertal growth spurt and be compensated by the greater energy and nutrient needs due to the intensive growth and development of the body [53]. Considering the results of other studies, the effect of disinhibition on body weight among adolescents was inconclusive. Similar to the present study, no significant correlation between uncontrolled eating and the BMI was found in adolescents from the UK [10] and with the BMI z-score among adolescent girls from the USA [29]. A study carried out among Chinese adolescents showed that external eating and emotional eating were negatively related to a higher BMI [31]. On the other hand, research conducted among Polish children and adolescents aged 8–16 years showed that uncontrolled eating (measured with the TFEQ-13) or external eating (measured with the DEBQ-C) were positively correlated with the BMI [12]. Moreover, higher uncontrolled eating was positively related to the prevalence of obesity [25]. A positive weak correlation of disinhibition with BMI z-scores was also found in adolescents from Canada, which was significant in the total sample and in boys [30].

### Strengths and Limitations

The strengths of this cross-sectional study include a large sample of adolescents aged 11–13 years (n = 1450), with a similar percentage of boys and girls. The study was carried out among primary school students from all macro-regions of the country, covering rural and urban areas. The data were collected by well-trained researchers using a validated questionnaire (SF-FFQ4PolishChildren^®^) [36], and the anthropometric measurements were taken using the same type of equipment across the academic centres in order to reduce inter-centre bias. The present study included both measures of adolescent body weight status, i.e., the BMI z-score and WHtR, while the vast majority of studies on children’s eating styles and their relationship with obesity have referred to the BMI only [10,12,29,30,31]. Next, two validated diet quality scores have been applied, and as such, a holistic approach is considered to have advantages over the assessment of single dietary characteristics (i.e., foods) in nutrition epidemiology [6,7]. Another strength of this study is the inclusion of a number of important factors (i.e., sex, age, place of residence, family affluence, nutrition knowledge, physical activity, screen time, and BMI z-score), the impact of which on the studied relationships was controlled in the logistic regression analysis. Furthermore, a variety of statistical methods were used to strengthen the conclusions. Therefore, this is a comprehensive study on the associations between problematic eating styles, diet quality, and body weight status among adolescents, the findings of which can, to some extent, be generalised to adolescents from other European countries.

There are several limitations of this study that should be highlighted for future research. Although the self-reported data were collected with the validated questionnaire, the possibility of social desirability bias cannot be excluded [54]. Psychological characteristics, such as social desirability, may affect the dietary reports of children and adolescents. They may be prone to underreport their consumption of unhealthy foods (e.g., snacks) and overreport their consumption of foods commonly known as healthy (e.g., fruit and vegetables). Future research should consider using precise methods of body composition assessment (e.g., Dual-Energy X-ray Absorptiometry, DXA; Bioelectrical Impedance Analysis, BIA) and taking into account other factors linked to the eating styles that were not included in the present study, such as social desirability [45], body image [52], psychological stress [15,18,31], and other psychological characteristics (e.g., anxiety and depression symptoms) [31] as well as the inclusion of genotype testing [51].

## 5. Conclusions

The findings suggest that both eating styles may be predictors of diet quality among adolescents, especially regarding unhealthy characteristics. Greater disinhibited eating was associated with a greater non-Healthy Diet Index, while greater restrained eating was associated with a lower non-Healthy Diet Index. It has been demonstrated that the pro-Healthy Diet Index was not significantly related to both problematic eating styles among adolescents. An adolescent’s body weight status was related to restrained eating. Adolescents with ‘higher RE & low DE’ were less likely to be underweight and more likely to be overweight and to have abdominal obesity. However, disinhibited eating was not significantly associated with adolescents’ body weight status. Further longitudinal studies are needed to assess the cause-and-effect relationship between eating styles, diet, and body weight status among adolescents.

## Figures and Tables

**Table 1:** Participant characteristics.

Variables	Total		Boys	Girls	*p*-Value
	n	%	%	%	
Sample size	1450		701	749	
Sample percentage		100.0	48.3	51.7	
Demographic, socioeconomic, and lifestyle factors					
Age ^1^, years (range: 11–13)	1450	11.9 (0.5)	11.9 (0.5)	11.9 (0.5)	0.880
Place of residence					0.922
rural	609	42.0	41.8	42.2	
urban	841	58.0	58.2	57.8	
FAS ^1^, points (range: 0–7)	1450	5.3 (1.5)	5.3 (1.5)	5.4 (1.5)	0.499
low	353	24.3	24.8	23.9	0.601
moderate	726	50.1	50.8	49.4	
high	371	25.6	24.4	26.7	
NK score ^1^, points (range: 0–18)	1450	6.1 (2.8)	5.7 (2.8)	6.4 (2.8)	<0.001
low	645	44.5	51.2	38.2	<0.001
moderate/high	805	55.5	48.8	61.8	
PA score ^1^, points (range: 0–5)	1450	3.7 (1.3)	3.8 (1.3)	3.6 (1.2)	0.010
low	138	9.5	9.6	9.5	<0.001
moderate	852	58.8	52.9	64.2	
high	460	31.7	37.5	26.3	
ST score ^1^, points (range: 0–5)	1450	0.9 (1.1)	1.0 (1.2)	0.7 (1.0)	<0.001
<2 h/day	677	46.7	42.5	50.6	<0.001
2 to <4 h/day	497	34.3	34.8	33.8	
≥4 h/day	276	19.0	22.7	15.6	
Eating styles points (range: 0–100)					
restrained eating (RE) ^1^	1450	48.2 (21.9)	47.6 (22.7)	48.7 (21.0)	0.483
disinhibited eating (DE) ^1^	1450	31.5 (16.8)	31.3 (17.1)	31.7 (16.5)	0.664
Restrained and disinhibited eating levels					
low RE and DE	356	24.6	24.1	25.0	0.687
higher RE and low DE	386	26.6	28.0	25.4	
low RE and higher DE	400	27.6	27.5	27.6	
higher RE and DE	308	21.2	20.4	22.0	
Diet quality					
pHDI ^1^, points (range: 0–100)	1450	28.4 (14.6)	27.5 (14.6)	29.2 (14.6)	0.007
bottom tertile	483	33.3	36.2	30.6	0.026
middle tertile	480	33.1	33.2	33.0	
upper tertile	487	33.6	30.5	36.4	
nHDI ^1^, points (range: 0–100)	1450	14.0 (11.5)	15.0 (12.3)	13.1 (10.6)	<0.001
bottom tertile	464	32.0	29.2	34.6	<0.001
middle tertile	488	33.7	31.2	35.9	
upper tertile	498	34.3	39.5	29.5	
Body weight status					
BMI z-score ^1^	1450	0.39 (1.07)	0.38 (1.04)	0.40 (1.10)	0.817
BMI categories					
underweight (<−1 SD)	63	4.3	3.0	5.6	0.044
normal weight (−1 SD ÷ 1 SD)	1037	71.5	73.2	70.0	
overweight (>1 SD)	350	24.1	23.8	24.4	
WHtR ^1^	1450	0.43 (0.06)	0.44 (0.06)	0.42 (0.05)	<0.001
abdominal obesity	178	12.3	16.3	8.5	<0.001

^1^ mean (standard deviation, SD); FAS, family affluence scale; NK, nutrition knowledge; PA, physical activity; ST, screen time; pHDI, pro-Healthy Diet Index; nHDI, non-Healthy Diet Index; BMI, body mass index; WHtR, waist-to-height ratio; *p*-value, significance level of the Mann–Whitney test (continuous variables) or chi^2^ test, with Yates’ correction if necessary (categorical variables).

**Table 2:** Spearman’s correlation coefficients between eating styles and socioeconomic and lifestyle factors, diet quality, and body weight measures among Polish adolescents (n = 1450).

Variables	Restrained Eating (RE)		Disinhibited Eating (DE)	
	r	*p*-Value	r	*p*-Value
Eating styles				
RE	-			
DE	−0.137	<0.001	-	
Socioeconomic and lifestyle factors				
FAS	0.070	0.007	−0.041	0.115
NK score	0.050	0.057	−0.073	0.005
PA score	0.111	<0.001	−0.138	<0.001
ST score	−0.071	0.007	0.197	<0.001
Diet quality scores				
pHDI	0.037	0.158	−0.051	0.053
nHDI	−0.178	<0.001	0.232	<0.001
Body weight measures				
BMI z-score	0.253	<0.001	−0.036	0.168
WHtR	0.197	<0.001	−0.025	0.344

r, Spearman’s correlation coefficient; FAS, family affluence scale; NK, nutrition knowledge; PA, physical activity; ST, screen time; pHDI, pro-Healthy Diet Index; nHDI, non-Healthy Diet Index; BMI, body mass index; WHtR, waist-to-height ratio.

**Table 3:** Diet quality and body weight status across the restrained and disinhibited eating levels among Polish adolescents (n = 1450).

Variables	Total	Low RE and DE	Higher RE and Low DE	Low RE and Higher DE	Higher RE and DE	*p*-Value
	%	%	%	%	%	
Sample size	1450	356	386	400	308	
Sample percentage	100.0	24.6	26.6	27.6	21.2	
Diet quality						
pHDI ^1^, points (range: 0–100)	28.4 (14.6)	28.8 (15.5)	29.4 (14.6)	27.5 (13.8)	27.9 (14.6)	0.351
bottom tertile	33.3	36.8	29.0	33.3	34.7	0.213
middle tertile	33.1	30.1	35.5	35.8	30.2	
upper tertile	33.6	33.1	35.5	31.0	35.1	
pHDI components ^1^, times/day (range: 0–2)						
vegetables	0.64 (0.49)	0.70 (0.54) ^a^	0.65 (0.48)	0.58 (0.46) ^a^	0.64 (0.49)	0.034
fruit	0.69 (0.55)	0.67 (0.56)	0.77 (0.58) ^a^	0.68 (0.53)	0.65 (0.54) ^a^	0.016
dairy products	0.78 (0.51)	0.79 (0.49)	0.76 (0.48)	0.78 (0.52)	0.77 (0.55)	0.250
fish	0.16 (0.24)	0.14 (0.22)	0.17 (0.24)	0.15 (0.24)	0.17 (0.24)	0.049
nHDI ^1^, points (range: 0–100)	14.0 (11.5)	13.8 (11.1) ^a,b^	10.6 (8.7) ^a,c,d^	17.4 (13.6) ^b,c,e^	14.0 (10.6) ^d,e^	<0.001
bottom tertile	32.0	34.3	40.7	22.5	30.8	<0.001
middle tertile	33.7	31.7	36.8	31.5	34.7	
upper tertile	34.3	34.0	22.5	46.0	34.4	
nHDI components ^1^, times/day (range: 0–2)						
fast food	0.13 (0.21)	0.12 (0.19) ^a^	0.08 (0.12) ^b,c^	0.17 (0.28) ^a,b^	0.14 (0.21) ^c^	<0.001
sweets	0.52 (0.45)	0.52 (0.40) ^a,b^	0.41 (0.38) ^a,c,d^	0.65 (0.52) ^b,c,e^	0.49 (0.42) ^d,e^	<0.001
sweetened beverages	0.42 (0.46)	0.42 (0.48) ^a^	0.33 (0.38) ^b,c^	0.51 (0.52) ^a,b^	0.43 (0.44) ^c^	<0.001
energy drinks	0.05 (0.17)	0.04 (0.16)	0.03 (0.12) ^a,b^	0.06 (0.21) ^a^	0.06 (0.16) ^b^	<0.001
Body weight status						
BMI z-score ^1^	0.39 (1.07)	0.17 (1.02) ^a,b^	0.63 (1.04) ^a,c^	0.24 (1.10) ^c,d^	0.53 (1.05) ^b,d^	<0.001
underweight (<−1 SD)	4.3	6.5	1.0	6.0	3.9	<0.001
normal weight (−1 SD ÷ 1 SD)	71.5	75.3	68.9	74.0	67.2	
overweight (>1 SD)	24.1	18.3	30.1	20.0	28.9	
WHtR ^1^	0.43 (0.06)	0.42 (0.05) ^a,b^	0.44 (0.06) ^a,c^	0.42 (0.05) ^c,d^	0.44 (0.06) ^b,d^	<0.001
abdominal obesity (≥0.5)	12.3	10.4	15.5	9.8	13.6	0.048

^1^ mean (standard deviation, SD); pHDI, pro-Healthy Diet Index; nHDI, non-Healthy Diet Index; BMI, body mass index; WHtR, waist-to-height ratio; *p*-value, significance level of the Kruskal–Wallis test (for continuous variables) or chi^2^ test (for categorical variables); ^a-a, b-b, c-c, d-d, e-e^ the same letters in superscripts indicate significant difference between groups.

**Table 4:** Odds ratios (95% CI) for the diet quality and body weight status by the restrained and disinhibited eating levels among Polish adolescents (n = 1450).

Variables	Low RE and DE	Higher RE and Low DE		Low RE and Higher DE		Higher RE and DE	
	Ref.	OR_crude_	OR_adj_	OR_crude_	OR_adj_	OR_crude_	OR_adj_
Sample size	356	386		400		308	
Diet quality							
pHDI (ref.: bottom tertile)							
middle tertile	1.00	1.50 (1.05; 2.14)	1.46 (1.00; 2.14)	1.32 (0.93; 1.87)	1.41 (0.98; 2.02)	1.06 (0.73; 1.56)	1.09 (0.74; 1.62)
upper tertile	1.00	1.36 (0.95; 1.93)	1.13 (0.76; 1.69)	1.04 (0.73; 1.46)	1.28 (0.88; 1.87)	1.12 (0.78; 1.62)	1.06 (0.71; 1.59)
nHDI (ref.: bottom tertile)							
middle tertile	1.00	0.98 (0.69; 1.39)	1.12 (0.77; 1.62)	1.51 (1.04; 2.20)	1.49 (1.02; 2.19)	1.22 (0.83; 1.77)	1.24 (0.84; 1.84)
upper tertile	1.00	0.56 (0.39; 0.80)	0.67 (0.44; 1.00)	2.06 (1.44; 2.95)	1.90 (1.29; 2.81)	1.13 (0.77; 1.64)	1.19 (0.79; 1.79)
Body weight status							
BMI z-score (ref.: normal weight)							
underweight (<−1 SD)	1.00	0.18 (0.06; 0.51)	0.17 (0.06; 0.49)	0.94 (0.52; 1.71)	0.94 (0.50; 1.73)	0.68 (0.33; 1.39)	0.67 (0.32; 1.39)
overweight (>1 SD)	1.00	1.80 (1.27; 2.55)	2.02 (1.41; 2.91)	1.11 (0.77; 1.61)	0.96 (0.66; 1.40)	1.77 (1.23; 2.56)	1.73 (1.19; 2.52)
WHtR (ref.: lack of abdominal obesity)							
abdominal obesity (≥0.5)	1.00	1.59 (1.02; 2.46)	1.79 (1.13; 2.82)	0.93 (0.58; 1.50)	0.77 (0.47; 1.27)	1.36 (0.85; 2.18)	1.31 (0.81; 2.14)

CI, confidence interval; OR_crude_, odds ratio not adjusted; OR_adj_, odds ratios adjusted for the confounders: sex, age, place of residence, family affluence scale (FAS), nutrition knowledge (NK), physical activity (PA), screen time (ST), and BMI z-score (in the analyses for pHDI and nHDI only); pHDI, pro-Healthy Diet Index; nHDI, non-Healthy Diet Index; BMI, body mass index; WHtR, waist-to-height ratio.

## Data Availability

The raw data supporting the conclusions of this article will be made available from the corresponding author upon reasonable request.

## Supplementary Materials

The following supporting information can be downloaded at: https://www.mdpi.com/article/10.3390/nu16213601/s1, Table S1: Participant characteristics by the restrained and disinhibited eating levels (n = 1450); Table S2: Difference in eating styles across demographic, socioeconomic, and lifestyle factors, diet quality, and body weight measures among Polish adolescents (n = 1450).

## References

[1] World Health Organization (WHO) (2024). Obesity and Overweight. Fact Sheets. https://www.who.int/news-room/fact-sheets/detail/obesity-and-overweight.

[2] World Health Organization (WHO) (2024). Body Mass Index Among Children. https://www.who.int/data/gho/data/themes/topics/indicator-groups/indicator-group-details/GHO/bmi-among-children.

[3] Sina E., Boakye D., Christianson L., Ahrens W., Hebestreit A. (2022). Social Media and Children’s and Adolescents’ Diets: A Systematic Review of the Underlying Social and Physiological Mechanisms. Adv. Nutr..

[4] Moore Heslin A., McNulty B. (2023). Adolescent nutrition and health: Characteristics, risk factors and opportunities of an overlooked life stage. Proc. Nutr. Soc..

[5] Vidal R., Rivera-Navarro J., Gravina L., Díez J., Franco M. (2024). Correlates of eating behaviors in adolescence: A systematic review of qualitative studies. Nutr. Rev..

[6] Waijers P.M.C.M., Feskens E.J.M., Ocké M.C. (2007). A critical review of pre-defined diet quality scores. Br. J. Nutr..

[7] Wadolowska L., Kowalkowska J., Czarnocinska J., Jezewska-Zychowicz M., Babicz-Zielinska E. (2017). Comparing dietary patterns derived by two methods and their associations with obesity in Polish girls aged 13-21 years: The cross-sectional GEBaHealth study. Perspect. Public Health.

[8] Wingrove K., Lawrence M.A., McNaughton S.A. (2022). A Systematic Review of the Methods Used to Assess and Report Dietary Patterns. Front. Nutr..

[9] Johnson L., Mander A.P., Jones L.R., Emmett P.M., Jebb S.A. (2008). Energy-dense, low-fiber, high-fat dietary pattern is associated with increased fatness in childhood. Am. J. Clin. Nutr..

[10] Booth C., Spronk D., Grol M., Fox E. (2018). Uncontrolled eating in adolescents: The role of impulsivity and automatic approach bias for food. Appetite.

[11] Bryant E.J., Rehman J., Pepper L.B., Walters E.R. (2019). Obesity and Eating Disturbance: The Role of TFEQ Restraint and Disinhibition. Curr. Obes. Rep..

[12] Czepczor-Bernat K., Brytek-Matera A. (2019). Children’s and Mothers’ Perspectives of Problematic Eating Behaviours in Young Children and Adolescents: An Exploratory Study. Int. J. Environ. Res. Public Health.

[13] Braet C., Claus L., Goossens L., Moens E., Van Vlierberghe L., Soetens B. (2008). Differences in eating style between overweight and normal-weight youngsters. J. Health Psychol..

[14] Małachowska A., Gębski J., Jeżewska-Zychowicz M. (2023). Childhood Food Experiences and Selected Eating Styles as Determinants of Diet Quality in Adulthood-A Cross-Sectional Study. Nutrients.

[15] Hagerman C.J., Stock M.L., Beekman J.B., Yeung E.W., Persky S. (2021). The ironic effects of dietary restraint in situations that undermine self-regulation. Eat. Behav..

[16] Schaumberg K., Anderson D.A., Anderson L.M., Reilly E.E., Gorrell S. (2016). Dietary restraint: What’s the harm? A review of the relationship between dietary restraint, weight trajectory and the development of eating pathology. Clin. Obes..

[17] Contento I.R., Zybert P., Williams S.S. (2005). Relationship of cognitive restraint of eating and disinhibition to the quality of food choices of Latina women and their young children. Prev. Med..

[18] Herhaus B., Petrowski K. (2021). The effect of restrained eating on acute stress-induced food intake in people with obesity. Appetite.

[19] van Strien T., Herman C.P., Verheijden M.W. (2014). Dietary restraint and body mass change. A 3-year follow up study in a representative Dutch sample. Appetite.

[20] Favieri F., Marini A., Casagrande M. (2021). Emotional Regulation and Overeating Behaviors in Children and Adolescents: A Systematic Review. Behav. Sci..

[21] van Strien T., Cebolla A., Etchemendy E., Gutiérrez-Maldonado J., Ferrer-García M., Botella C., Baños R. (2013). Emotional eating and food intake after sadness and joy. Appetite.

[22] Ljubičić M., Matek Sarić M., Klarin I., Rumbak I., Colić Barić I., Ranilović J., Dželalija B., Sarić A., Nakić D., Djekic I. (2023). Emotions and Food Consumption: Emotional Eating Behavior in a European Population. Foods.

[23] Kakoschke N., Kemps E., Tiggemann M. (2015). External eating mediates the relationship between impulsivity and unhealthy food intake. Physiol. Behav..

[24] Stroebele N., De Castro J.M. (2004). Effect of ambience on food intake and food choice. Nutrition.

[25] Czepczor-Bernat K., Brytek-Matera A., Matusik P. (2020). The Homeostatic Theory of Obesity: An Empirical Verification of the Circle of Discontent with an Assessment of Its Relationship to Restrained and Uncontrolled Eating among Children and Adolescents. Int. J. Environ. Res. Public Health.

[26] Dzielska A., Mazur J., Małkowska-Szkutnik A., Kołoło H. (2009). Adaptation of the Three-Factor Eating Questionnaire (TFEQ-13) for school-aged adolescents in a population study. Probl. Hig. Epidemiol..

[27] Mazur J., Dzielska A., Małkowska-Szkutnik A. (2011). Psychological determinants of selected eating behaviours in adolescents. Med. Wieku Rozwoj..

[28] Bryant E.J., Thivel D., Chaput J.P., Drapeau V., Blundell J.E., King N.A. (2018). Development and validation of the Child Three-Factor Eating Questionnaire (CTFEQr17). Public Health Nutr..

[29] Banna J.C., Panizza C.E., Boushey C.J., Delp E.J., Lim E. (2018). Association between Cognitive Restraint, Uncontrolled Eating, Emotional Eating and BMI and the Amount of Food Wasted in Early Adolescent Girls. Nutrients.

[30] Gallant A.R., Tremblay A., Pérusse L., Bouchard C., Després J.P., Drapeau V. (2010). The Three-Factor Eating Questionnaire and BMI in adolescents: Results from the Québec family study. Br. J. Nutr..

[31] Hou F., Xu S., Zhao Y., Lu Q., Zhang S., Zu P., Sun Y., Su P., Tao F. (2013). Effects of emotional symptoms and life stress on eating behaviors among adolescents. Appetite.

[32] de Lauzon B., Romon M., Deschamps V., Lafay L., Borys J.M., Karlsson J., Ducimetière P., Charles M.A., Fleurbaix Laventie Ville Sante Study Group (2004). The Three-Factor Eating Questionnaire-R18 is able to distinguish among different eating patterns in a general population. J. Nutr..

[33] Elfhag K., Tholin S., Rasmussen F. (2008). Consumption of fruit, vegetables, sweets and soft drinks are associated with psychological dimensions of eating behaviour in parents and their 12-year-old children. Public Health Nutr..

[34] Shriver L.H., Dollar J.M., Calkins S.D., Keane S.P., Shanahan L., Wideman L. (2020). Emotional Eating in Adolescence: Effects of Emotion Regulation, Weight Status and Negative Body Image. Nutrients.

[35] Hamulka J., Wadolowska L., Hoffmann M., Kowalkowska J., Gutkowska K. (2018). Effect of an Education Program on Nutrition Knowledge, Attitudes toward Nutrition, Diet Quality, Lifestyle, and Body Composition in Polish Teenagers. The ABC of Healthy Eating Project: Design, Protocol, and Methodology. Nutrients.

[36] Kowalkowska J., Wadolowska L., Hamulka J., Wojtas N., Czlapka-Matyasik M., Kozirok W., Bronkowska M., Sadowska J., Naliwajko S., Dziaduch I. (2019). Reproducibility of a Short-Form, Multicomponent Dietary Questionnaire to Assess Food Frequency Consumption, Nutrition Knowledge, and Lifestyle (SF-FFQ4PolishChildren) in Polish Children and Adolescents. Nutrients.

[37] Karlsson J., Persson L.O., Sjöström L., Sullivan M. (2000). Psychometric properties and factor structure of the Three-Factor Eating Questionnaire (TFEQ) in obese men and women. Results from the Swedish Obese Subjects (SOS) study. Int. J. Obes. Relat. Metab. Disord..

[38] Stewart A., Marfell-Jones M.J., International Society for the Advancement of Kinanthropometry, International Society for the Advancement of Kinanthropometry (2011). International Standards for Anthropometric Assessment.

[39] Kułaga Z., Różdżyńska A., Palczewska I., Grajda A., Gurzkowska B., Napieralska E., Litwin M., Grupa Badaczy OLAF (2010). Percentile charts of height, body mass and body mass index in children and adolescents in Poland—results of the OLAF study. Stand. Med. Pediatr..

[40] Ashwell M., Gunn P., Gibson S. (2012). Waist-to-height ratio is a better screening tool than waist circumference and BMI for adult cardiometabolic risk factors: Systematic review and meta-analysis. Obes. Rev..

[41] Mazur J. (2013). Family Affluence Scale—Validation study and suggested modification. Hygeia Pub. Health.

[42] Górnicka M., Hamulka J., Wadolowska L., Kowalkowska J., Kostyra E., Tomaszewska M., Czeczelewski J., Bronkowska M. (2020). Activity–Inactivity Patterns, Screen Time, and Physical Activity: The Association with Overweight, Central Obesity and Muscle Strength in Polish Teenagers. Report from the ABC of Healthy Eating Study. Int. J. Environ. Res. Public Health.

[43] Stanisz A. (2006). The Accessible Course of Statistics with the Use of STATISTICA PL Using Examples from Medicine.

[44] de Lauzon-Guillain B., Romon M., Musher-Eizenman D., Heude B., Basdevant A., Charles M.A., Fleurbaix-Laventie Ville Santé Study Group (2009). Cognitive restraint, uncontrolled eating and emotional eating: Correlations between parent and adolescent. Matern. Child. Nutr..

[45] Kowalkowska J., Poínhos R. (2021). Eating Behaviour among University Students: Relationships with Age, Socioeconomic Status, Physical Activity, Body Mass Index, Waist-to-Height Ratio and Social Desirability. Nutrients.

[46] Löffler A., Luck T., Then F.S., Luck-Sikorski C., Pabst A., Kovacs P., Böttcher Y., Breitfeld J., Tönjes A., Horstmann A. (2017). Effects of psychological eating behaviour domains on the association between socio-economic status and BMI. Public Health Nutr..

[47] Pigeyre M., Rousseaux J., Trouiller P., Dumont J., Goumidi L., Bonte D., Dumont M.P., Chmielewski A., Duhamel A., Amouyel P. (2016). How obesity relates to socio-economic status: Identification of eating behavior mediators. Int. J. Obes..

[48] Bond M.J., McDowell A.J., Wilkinson J.Y. (2001). The measurement of dietary restraint, disinhibition and hunger: An examination of the factor structure of the Three Factor Eating Questionnaire (TFEQ). Int. J. Obes. Relat. Metab. Disord..

[49] Alberga A.S., Edache I.Y., Sigal R.J., von Ranson K.M., Russell-Mayhew S., Kenny G.P., Doucette S., Prud’homme D., Hadjiyannakis S., Cameron J.D. (2022). Effects of the HEARTY exercise randomized controlled trial on eating behaviors in adolescents with obesity. Obes. Sci. Pract..

[50] Saat N.Z.M., Hanawi S.A., Chew N.H.H., Ahmad M., Farah N.M.F., Kadar M., Yahya H.M., Warif N.M.A., Daud M.K.M. (2023). The Association of Eating Behaviour with Physical Activity and Screen Time among Adolescents in the Klang Valley, Malaysia: A Cross-Sectional Study. Healthcare.

[51] Bryant E.J., King N.A., Blundell J.E. (2008). Disinhibition: Its effects on appetite and weight regulation. Obes. Rev..

[52] Megalakaki O., Mouveaux M., Hubin-Gayte M., Wypych L. (2013). Body image and cognitive restraint are risk factors for obesity in French adolescents. Eat. Weight. Disord..

[53] Shomaker L.B., Tanofsky-Kraff M., Savastano D.M., Kozlosky M., Columbo K.M., Wolkoff L.E., Zocca J.M., Brady S.M., Yanovski S.Z., Crocker M.K. (2010). Puberty and observed energy intake: Boy, can they eat!. Am. J. Clin. Nutr..

[54] Baxter S.D., Smith A.F., Litaker M.S., Baglio M.L., Guinn C.H., Shaffer N.M. (2004). Children’s Social Desirability and Dietary Reports. J. Nutr. Educ. Behav..

