# Supplementary material for: Restrained Eating and Disinhibited Eating: Association with Diet Quality and Body Weight Status Among Adolescents
Source: Nutrients. 2024 Oct 23;16(21):3601. doi: 10.3390/nu16213601 (PMC11547977; doi:10.3390/nu16213601)
Supplement: Supplementary file 1 [file nutrients-16-03601-s001.zip › nutrients-3245077-supplementary.pdf]

nutrients MDPI

## Supplementary Materials

# Restrained Eating and Disinhibited Eating: Association with Diet Quality and Body Weight Status Among Adolescents

**Joanna Kowalkowska [1], Jadwiga Hamulka [2,*], Lidia Wadolowska [1], Magdalena Górnicka [2], Ewa Czarniecka-Skubina [3], Krystyna Gutkowska [4]**

[1] Department of Human Nutrition, Faculty of Food Science, University of Warmia and Mazury in Olsztyn, 45F Sloneczna Street, 10-718 Olsztyn, Poland; joanna.kowalkowska@uwm.edu.pl (J.K.), lidia.wadolowska@uwm.edu.pl (L.W.)

[2] Department of Human Nutrition, Institute of Human Nutrition Sciences, Warsaw University of Life Sciences (SGGW-WULS), 166 Nowoursynowska Street, 02-787 Warsaw, Poland; jadwiga_hamulka@sggw.edu.pl (J.H.), magdalena_gornicka@sggw.edu.pl (M.G.)

[3] Department of Food Gastronomy and Food Hygiene, Institute of Human Nutrition Sciences, Warsaw University of Life Sciences (SGGW-WULS), 166 Nowoursynowska Street, 02-787 Warsaw, Poland; ewa_czarniecka-skubina@sggw.edu.pl (E.C-S.)

[4] Department of Food Market and Consumption Research, Institute of Human Nutrition Sciences, Warsaw University of Life Sciences (SGGW-WULS), 166 Nowoursynowska Street, 02-787 Warsaw, Poland; krystyna_gutkowska@sggw.edu.pl (K.G.)

**Table S1.** Participant characteristics by the restrained and disinhibited eating levels (*n* = 1450)

| Variables | Total | | Low RE & DE | Higher RE & low DE | Low RE & higher DE | Higher RE & DE | p-value |
|---|---|---|---|---|---|---|---|
| | *n* | % | % | % | % | % | |
| Sample size | 1450 | | 356 | 386 | 400 | 308 | |
| Sample percentage | | 100.0 | 24.6 | 26.6 | 27.6 | 21.2 | |
| Sex | 1450 | | | | | | |
| boys | 701 | 48.3 | 47.5 | 50.8 | 48.3 | 46.4 | 0.687 |
| girls | 749 | 51.7 | 52.5 | 49.2 | 51.8 | 53.6 | |
| Age[1], years | 1450 | 11.9 (0.5) | 12.0 (0.5) | 11.9 (0.5) | 12.0 (0.5) | 11.9 (0.5) | 0.430 |
| Eating styles, points[1] (range: 0-100) | | | | | | | |
| restrained eating (RE) | 1450 | 48.2 (21.9) | 30.7 (13.5)[a,b] | 68.8 (11.9)[a,c] | 31.1 (12.7)[c,d] | 64.9 (10.3)[b,d] | < 0.001 |
| disinhibited eating (DE) | 1450 | 31.5 (16.8) | 19.4 (8.3)[a,b] | 17.7 (8.5)[c,d] | 45.7 (11.8)[a,c] | 44.4 (11.9)[b,d] | < 0.001 |
| Place of residence | 1450 | | | | | | |
| rural | 609 | 42.0 | 42.1 | 45.1 | 37.3 | 44.2 | 0.122 |
| urban | 841 | 58.0 | 57.9 | 54.9 | 62.8 | 55.8 | |

| Variables | Total | | Low RE & DE | Higher RE & low DE | Low RE & higher DE | Higher RE & DE | p-value |
|---|---|---|---|---|---|---|---|
| | *n* | % | % | % | % | % | |
| FAS, points[1] (range: 0-7) | 1450 | 5.3 (1.5) | 5.3 (1.6) | 5.5 (1.4)[a] | 5.2 (1.6)[a] | 5.4 (1.5) | 0.025 |
| low | 353 | 24.3 | 23.6 | 21.2 | 28.5 | 23.7 | 0.140 |
| moderate | 726 | 50.1 | 50.8 | 51.0 | 50.3 | 47.7 | |
| high | 371 | 25.6 | 25.6 | 27.7 | 21.3 | 28.6 | |
| Nutrition knowledge, points[1] (range: 0-18) | 1450 | 6.1 (2.8) | 6.2 (2.9) | 6.3 (2.8)[a] | 5.7 (2.8)[a] | 6.1 (2.8) | 0.023 |
| low | 645 | 44.5 | 41.6 | 40.7 | 49.8 | 45.8 | 0.042 |
| moderate/high | 805 | 55.5 | 58.4 | 59.3 | 50.3 | 54.2 | |
| Physical activity, points[1] (range: 0-5) | 1450 | 3.7 (1.3) | 3.7 (1.2)[a] | 3.9 (1.1)[b] | 3.4 (1.4)[a,b] | 3.7 (1.3) | < 0.001 |
| low | 138 | 9.5 | 7.6 | 5.4 | 15.3 | 9.4 | < 0.001 |
| moderate | 852 | 58.8 | 62.4 | 56.5 | 58.0 | 58.4 | |
| high | 460 | 31.7 | 30.1 | 38.1 | 26.8 | 32.1 | |
| Screen time, points[1] (range: 0-5) | 1450 | 0.9 (1.1) | 0.8 (1.1)[a] | 0.6 (1.0)[a,b,c] | 1.0 (1.2)[b] | 0.9 (1.1)[c] | < 0.001 |
| < 2 h/day | 677 | 46.7 | 48.3 | 57.8 | 38.8 | 41.2 | < 0.001 |
| 2 to < 4 h/day | 497 | 34.3 | 32.9 | 30.8 | 37.5 | 36.0 | |
| ≥ 4 h/day | 276 | 19.0 | 18.8 | 11.4 | 23.8 | 22.7 | |

[1]mean (standard deviation, SD); FAS, family affluence scale; p-value, significance level of the Kruskal-Wallis test (for continuous variables) or chi$^2$ test (for categorical variables); [a-a, b-b, c-c, d-d] the same letters in superscripts indicate significant difference between groups.

nutrients

MDPI

**Table S2.** Difference in eating styles across demographic, socioeconomic and lifestyle factors, diet quality, and body weight measures among Polish adolescents (*n* = 1450)

| Variables | Total | Restrained eating (RE)[1] | Disinhibited eating (DE)[1] |
|---|---|---|---|
| | *n* | mean (SD) | mean (SD) |
| Demographic, socioeconomic and lifestyle factors | | | |
| Sex | 1450 | | |
| boys | 701 | 47.6 (22.7) | 31.3 (17.1) |
| girls | 749 | 48.7 (21.0) | 31.7 (16.5) |
| p-value | | 0.483 | 0.664 |
| Place of residence | 1450 | | |
| rural | 609 | 49.6 (22.4) | 30.4 (16.2) |
| urban | 841 | 47.2 (21.4) | 32.3 (17.2) |
| p-value | | 0.048 | 0.057 |
| FAS | 1450 | | |
| low | 353 | 46.3 (22.0) | 33.3 (17.4) |
| moderate | 726 | 48.4 (21.5) | 31.1 (16.6) |
| high | 371 | 49.7 (22.4) | 30.7 (16.4) |
| p-value | | 0.075 | 0.075 |
| Nutrition knowledge | 1450 | | |
| low | 645 | 47.5 (21.3) | 32.7 (17.0) |
| moderate/high | 805 | 48.8 (22.3) | 30.5 (16.6) |
| p-value | | 0.241 | 0.010 |
| Physical activity | 1450 | | |
| low | 138 | 41.9 (21.0)[a,b] | 37.5 (17.6)[a,b] |
| moderate | 852 | 47.8 (20.9)[a] | 31.6 (16.1)[a,c] |
| high | 460 | 50.8 (23.4)[b] | 29.5 (17.3)[b,c] |
| p-value | | < 0.001 | < 0.001 |
| Screen time (hours/day) | 1450 | | |
| <2 | 677 | 49.6 (22.5)[a] | 28.3 (15.8)[a,b] |
| 2 to <4 | 497 | 47.9 (20.7) | 32.4 (15.7)[a,c] |
| ≥ 4 | 276 | 45.3 (22.2)[a] | 37.8 (18.9)[b,c] |
| p-value | | 0.025 | < 0.001 |
| Diet quality | | | |
| pHDI | 1450 | | |
| bottom tertile | 483 | 46.9 (20.9) | 31.9 (17.3) |
| middle tertile | 480 | 48.7 (21.7) | 32.5 (16.8) |
| upper tertile | 487 | 49.1 (22.8) | 30.1 (16.2) |
| p-value | | 0.173 | 0.189 |
| nHDI | 1450 | | |
| bottom tertile | 464 | 51.6 (22.2) [a] | 27.2 (16.1) [a,b] |
| middle tertile | 488 | 49.7 (21.2) [b] | 31.1 (15.5) [a,c] |
| upper tertile | 498 | 43.5 (21.4) [a,b] | 35.9 (17.6) [b,c] |
| p-value | | < 0.001 | < 0.001 |
| Body weight status | | | |
| BMI categories | 1450 | | |
| underweight (< -1 SD) | 63 | 35.4 (22.4)[a,b] | 32.5 (14.2) |
| normal weight (-1 SD ÷ 1 SD) | 1037 | 46.9 (22.0)[a,c] | 31.6 (16.7) |
| overweight (> 1 SD) | 350 | 54.3 (19.7 [b,c] | 31.2 (17.6) |
| p-value | | < 0.001 | 0.629 |
| WHtR categories | 1450 | | |
| lack of abdominal obesity (< 0.5) | 1272 | 47.4 (22.0) | 31.6 (16.5) |
| abdominal obesity (≥ 0.5) | 178 | 53.6 (19.8) | 31.1 (18.5) |
| p-value | | < 0.001 | 0.503 |

[1]range: 0-100 points; n – sample size; SD, standard deviation; FAS, family affluence scale; pHDI, pro-Healthy Diet Index; nHDI, non-Healthy Diet Index; BMI, body mass index; WHtR, waist-to-height ratio; p-value, significance level of the Mann-Whitney test (for comparison of 2 groups) or Kruskal-Wallis test (for 3 groups); [a-a, b-b, c-c] the same letters in superscripts indicate significant difference between groups (in columns).
